# Supplementary material for: Vitamin D Is Associated with Lipid Metabolism: A Sex- and Age-Dependent Analysis of a Large Outpatient Cohort
Source: Nutrients. 2024 Nov 18;16(22):3936. doi: 10.3390/nu16223936 (PMC11597382; doi:10.3390/nu16223936)
Supplement: Supplementary file 1 [file nutrients-16-03936-s001.zip › nutrients-3267390-supplementary.pdf]

Supplementary Figure S1 Correlation of vitamin D with lipids in people under 50 years of age

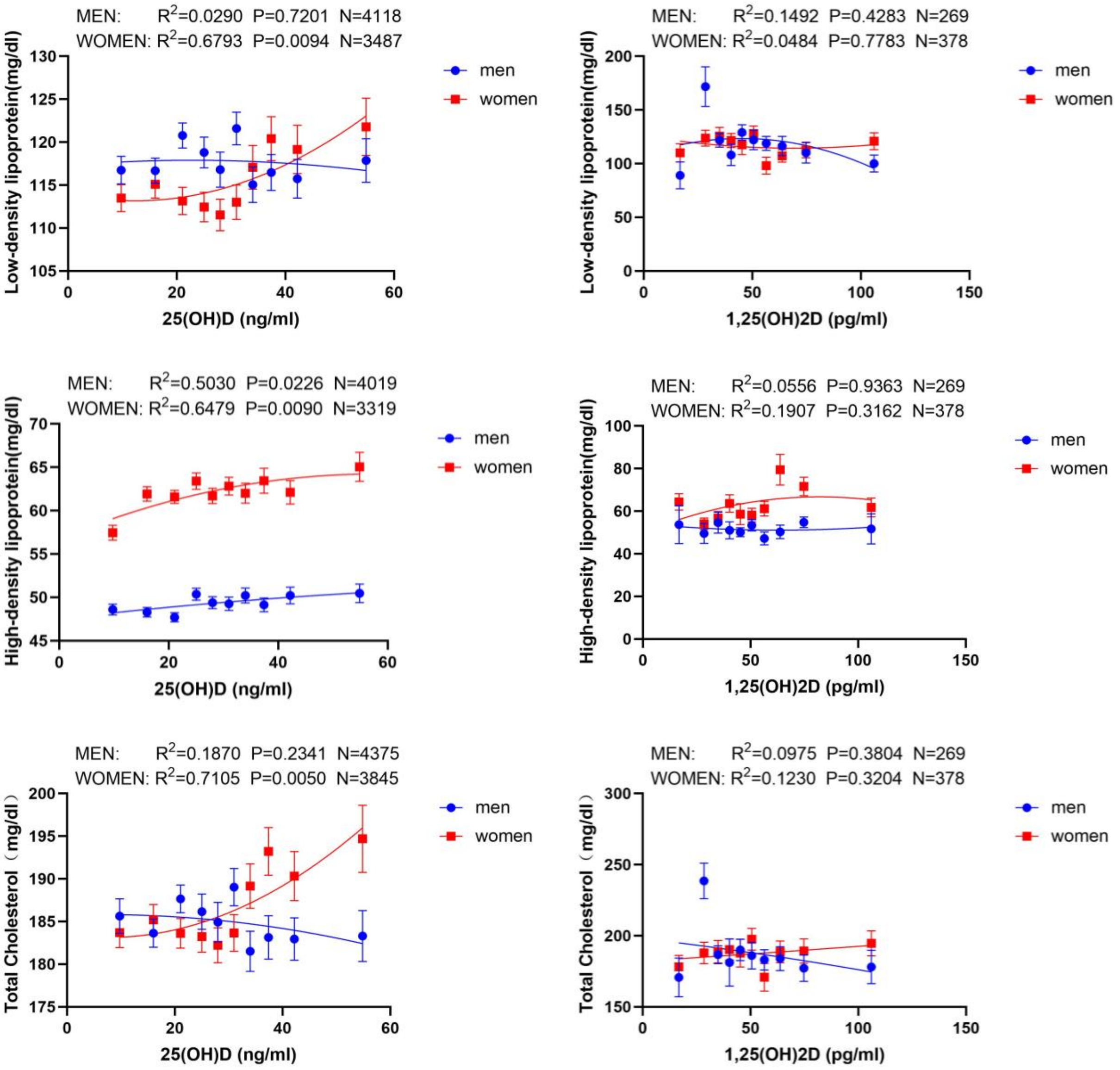

In the under-50 population, for 25(OH)D, women were positively associated with all three lipid parameters, whereas men were positively associated only with HDL. In contrast, 1,25(OH)$_2$D did not correlate with any of the lipids in people before 50 years of age.

Abbreviations: 25(OH)D: 25-hydroxy-vitamin D; 1,25(OH)2D: 1,25-dihydroxy-vitamin D.

Supplementary Figure S2 Correlation of vitamin D with lipids in people over 50 years of age

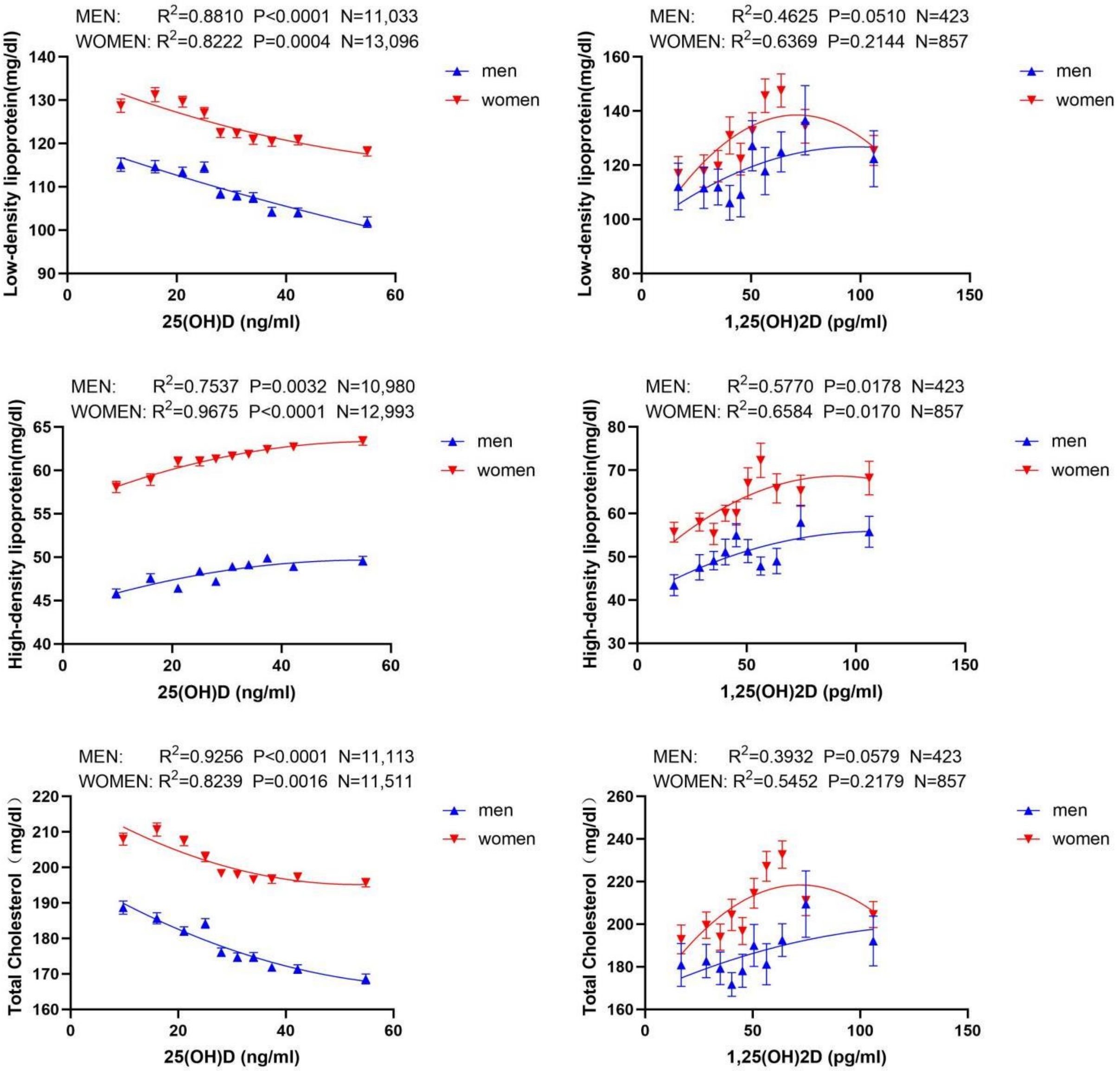

After the age of 50 years, for 25(OH)D, the correlations with LDL and TC reversed to negative in women and remained positive for HDL, whereas in men the correlations with the three lipid parameters were the same as in women. For 1,25(OH)$_2$D, after the age of 50 years, the correlation was positive only for HDL, and there were no significant correlations with LDL and TC in either sex.

Abbreviations: 25(OH)D: 25-hydroxy-vitamin D; 1,25(OH)2D: 1,25-dihydroxy-vitamin D.
